# Diagnostic Accuracy of the E-Plate Serum Antibody Test Kit in Detecting *Helicobacter pylori* Infection Among Japanese Children

**DOI:** 10.2188/jea.JE20130078

**Published:** 2014-01-05

**Authors:** Junko Ueda, Masumi Okuda, Takeshi Nishiyama, Yingsong Lin, Yoshihiro Fukuda, Shogo Kikuchi

**Affiliations:** 1 Department of Public Health, Aichi Medical University School of Medicine, Nagakute, Aichi, Japan; 2 Department of General Medicine and Community Health Science, Hyogo College of Medicine, Sasayama, Hyogo, Japan

**Keywords:** *Helicobacter pylori*, serologic test, validity, stool antigen test

## Abstract

**Background:**

A number of noninvasive diagnostic tests are available to detect *Helicobacter pylori* infection. Data on serologic testing of children are lacking, however, and thus it remains unclear whether the serology cutoff points used for adults are appropriate for children.

**Methods:**

Serum and stool samples were obtained from 73 children who visited 5 hospitals in Japan between March 1993 and December 2009. Analysis of stool samples was carried out using an *H pylori* stool antigen enzyme-linked immunosorbent assay (HpSA ELISA), and serum antibodies to *H pylori* were examined using an antibody determination kit (E-Plate Eiken *H pylori* antibody). The validity of the serologic test was evaluated based on its sensitivity, specificity, and receiver operating characteristics curve.

**Results:**

Of the 73 children included in this study, 34 were HpSA-positive and 39 were negative. Among the 34 HpSA-positive patients, 32 were IgG-positive and 2 were IgG-negative. Of the 39 patients who were HpSA-negative, 38 were IgG-negative and 1 was IgG-positive. The sensitivity, specificity, and positive likelihood ratio for IgG antibody testing were 91.2%, 97.4%, and 35.6, respectively, based on the recommended adult cutoff point of 10 U/ml. Among children, use of cutoff points in the range of 7 to 9 U/ml yielded optimal values for sensitivity and specificity, as well as a positive likelihood ratio.

**Conclusions:**

The performance of the E-plate *anti-H pylori* IgG antibody test was comparable to that of the stool antigen test and is therefore suitable for epidemiologic studies of *H pylori* infection in large samples.

## INTRODUCTION

*Helicobacter pylori *(*H pylori*) causes gastrointestinal diseases such as gastritis and peptic ulcer in adults and children.^1^^,^^2^ In addition, previous reports have linked *H pylori* infection with iron deficiency anemia and thrombocytopenic purpura in children.^3^^,^^4^ Although the prevalence of *H pylori* infection remains high among Japanese adults, a marked decrease has been observed among Japanese children.^5^^–^^7^ The exact route of *H pylori* transmission remains to be clarified; however, a widely held view is that the vast majority of new infections are acquired during early childhood.^8^

Numerous noninvasive diagnostic tests are available to detect *H pylori* infection, including serologic, stool antigen (HpSA), and ^13^C-urea breath testing.^9^^–^^11^ Each test has strengths and weaknesses in terms of diagnostic accuracy, performance characteristics for the various samples collected in epidemiologic studies, and rapidity as a bedside diagnostic test. The serologic test for *H pylori* infection can be easily done using stored sera in epidemiologic studies involving large samples; however, concerns regarding validity have been raised due to its lower sensitivity and specificity as compared with the stool antigen and urea breath tests.^12^ Furthermore, there are few data on serologic tests for children, and thus it remains unclear whether the serology cutoffs used for adults are applicable to children. Here, we used a commercially available ELISA kit (E-plate) to assess the utility of serologic testing for *H pylori* infection among Japanese children.

## METHODS

### Study population

Serum and stool samples were collected from 73 consecutive patients with dyspepsia (mean [SD] age, 6.3 [4.3] years) who visited 5 hospitals in the Kinki area of Japan between March 1993 and December 2009. Informed consent was obtained from the parents of the children. The study was approved by the Ethics Committee of Aichi Medical University.

### HpSA ELISA

The presence of *H pylori* was determined according to the result of a stool antigen test using HpSA ELISA (Meridian HpSA, TFB, Meridian, USA). Stool samples were analyzed using spectrophotometry (450/630). A value less than 0.10 indicated a negative result, 0.10 to 0.119 indicated an indeterminable result, and 0.12 or higher indicated a positive result, as specified in the manufacturer’s instructions.

### Microplate enzyme immunoassay

Serum and stool samples were stored at −80°C until the laboratory assay was performed. Serum antibodies to *H pylori* were examined using a microplate enzyme immunoassay (EIA) and an antibody determination kit (E-Plate Eiken *H pylori* antibody, Eiken Chemical Co., Ltd., Tokyo, Japan). All samples were analyzed according to the manufacturer’s instructions, and the cutoff point was set at 10 U/ml. All assays were performed by experimenters blinded to the clinical status of the patients.

### Statistical analysis

Logistic regression analysis was performed to examine the possible effects of sex and age on the serologic test. To assess the criterion validity of the serologic test, sensitivities, specificities, positive likelihood ratios, and negative likelihood ratios were estimated relative to the HpSA assay (the gold standard), across all possible cutoff values for the serologic test. To exclude the possible effects of maternal IgG antibody, we conducted additional analysis that excluded children younger than 1 year.

Receiver operating characteristics (ROC) analysis was also conducted using the HpSA assay as the gold standard. The 95% CI of the area under the ROC curve (AUC) was calculated using the bootstrap method with 10 000 bootstrap samples.

To compute the AUC with the bootstrap 95% CI, the R package pROC was used. To estimate the validity indices, eg, sensitivity and specificity, the R package Diagnosis Med was used. All analyses, except those noted above, were performed using R version 2.13.0 for Windows.^13^

## RESULTS

**Figure 1.**
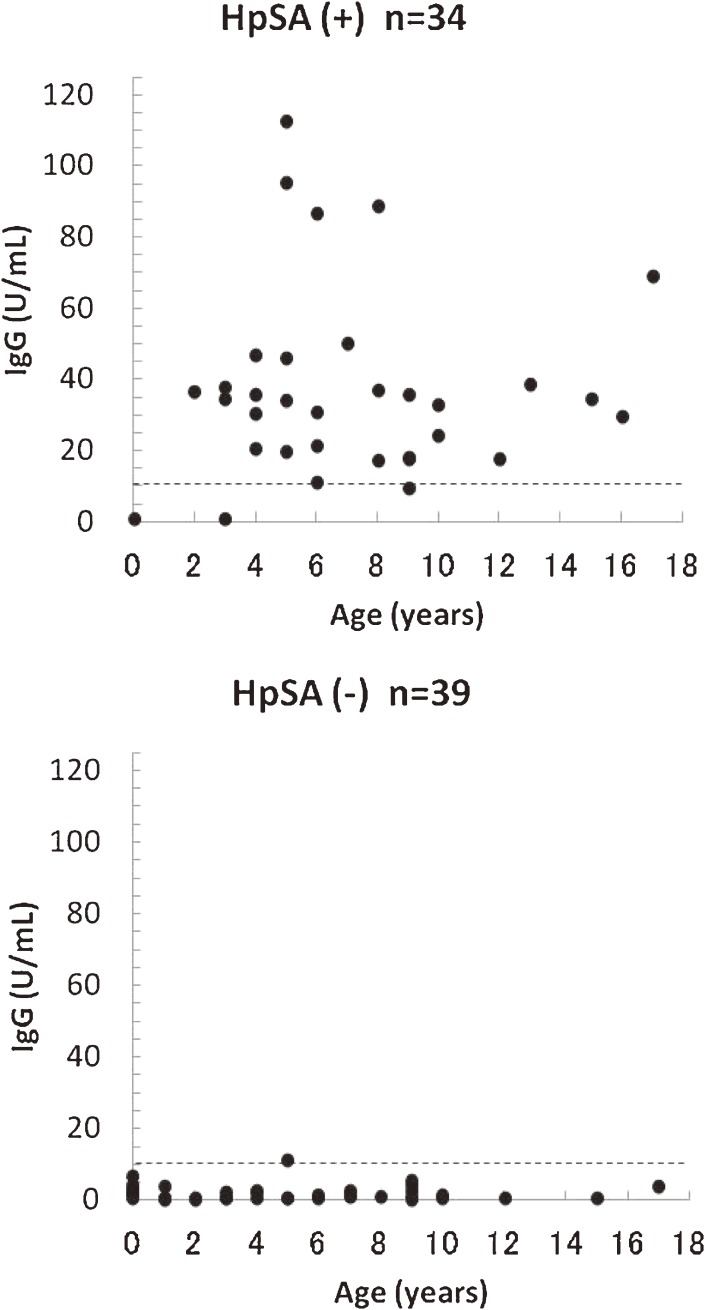
Results of the *H pylori* stool antigen (HpSA) assay and anti-*H pylori* IgG antibody test. (- - - -), cutoff value.

Of the 73 children included in this study, 34 were HpSA-positive and 39 were HpSA-negative (Figure 1). Of the 34 HpSA-positive patients, 32 were IgG-positive and 2 were IgG-negative. Of the 39 patients who were HpSA-negative, 38 were IgG-negative and 1 was IgG-positive.

Table 1 shows the age and sex distributions of the participants and the number of individuals and the test results of the HpSA and IgG antibody tests. Of the 9 children who were younger than 1 year, only 1 was HpSA-positive and IgG-negative.

**Table 1.** Characteristics of the study subjects

		HpSA(+)		HpSA(−)	
	
		IgG(+)	IgG(−)	IgG(+)	IgG(−)
Sex	Male	17	2	1	17
	Female	15	0	0	21

Age group	0	0	1	0	8
	1–5	12	1	1	14
	6–10	14	0	0	13
	10–17	6	0	0	3

HpSA: Stool antigen test; IgG; anti-*H pylori* IgG antibody test.

As shown in Table 2, when the cutoff point recommended by the manufacturer was used, the sensitivity, specificity, positive likelihood ratio, and negative likelihood ratio were 91.2%, 97.4%, 35.6, and 0.09, respectively. Using cutoff points in the range of 7 to 9 U/ml yielded optimal values for sensitivity, specificity, and positive likelihood ratio. In additional analysis that excluded the 9 children younger than 1 year, the results were similar, but the sensitivity was 97.0% when the cutoff point was in the range of 7 to 9 U/ml.

**Table 2.** Sensitivity and specificity of anti-*H pylori* IgG antibody test for Japanese children, by cutoff point

Cutoff	Sen (95% CI)	Spec (95% CI)	LR+ (95% CI)	LR− (95% CI)
3	94.12 (80.91–98.37)	79.49 (64.47–89.22)	4.59 (2.46–8.57)	0.07 (0.02–0.29)
4	94.12 (80.91–98.37)	87.18 (73.29–94.40)	7.34 (3.22–16.72)	0.07 (0.02–0.26)
5	94.12 (80.91–98.37)	92.31 (79.68–97.35)	12.24 (4.11–36.41)	0.06 (0.02–0.25)
6	94.12 (80.91–98.37)	94.87 (83.11–98.58)	18.35 (4.75–70.97)	0.06 (0.02–0.24)
7–9	94.12 (80.91–98.37)	97.44 (86.82–99.55)	36.71 (5.29–254.52)	0.06 (0.02–0.23)
10	91.18 (77.04–96.95)	97.44 (86.82–99.55)	35.56 (5.12–246.78)	0.09 (0.03–0.27)
11	88.24 (73.38–95.33)	97.44 (86.82–99.55)	34.41 (4.95–239.03)	0.12 (0.05–0.30)

Sen, sensitivity; Spec, specificity; LR, likelihood ratio.

**Figure 2.**
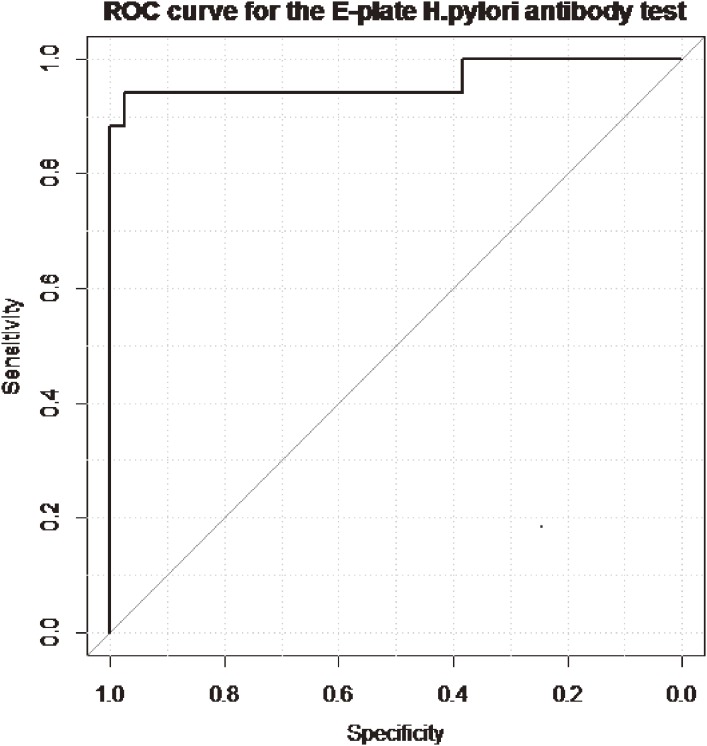
Receiver operating characteristics (ROC) curve for anti-*H pylori* IgG antibody test, with the HpSA assay as the gold standard.

Logistic regression analysis of the results of the HpSA assay revealed that neither sex (OR, 0.86; 95% CI, 0.07–10.3) nor age (OR, 0.94; 95% CI, 0.65–1.23) were significantly associated with presence of *H pylori*. Thus, all patients were included in the ROC analysis. The AUC for the anti-*H pylori* IgG antibody test was 0.96 (95% CI, 0.91–1.00; Figure 2).

## DISCUSSION

We compared the results of a serologic test and HpSA assay for *H pylori* infection in 73 Japanese children and found that the serologic test yielded excellent sensitivity and specificity, with the HpSA assay as the gold standard.

Previous studies reported mixed results regarding the utility of serologic tests for *H pylori* infection in children.^12^^,^^14^^–^^16^ A 2008 meta-analysis of 42 studies of children showed a sensitivity of 79.2% (95% CI, 77.3–81.0) and a specificity of 92.4% (95% CI, 91.6–93.3) for a serologic IgG antibody test.^11^ In general, serologic tests have high specificity but low sensitivity, especially among Japanese children younger than 10 years. One explanation for the low sensitivity in children is that production of antibodies to *H pylori* in children may differ from that in adults because the immunologic response is immature in children.^17^ Another explanation is that transfer of maternal IgG antibodies influences production of antibodies in response to *H pylori* infection in children.^18^

Because of the low sensitivity observed in serologic tests, clinical guidelines have recommended that tests based on the detection of serum antibodies against *H pylori* are not reliable in clinical settings.^19^ Despite the varied results of serologic tests, sensitivity and specificity were excellent for an ELISA kit using antigens derived from Japanese individuals. In addition, this laboratory-based serologic test is relatively inexpensive, and its accuracy is not affected by medication use.^20^ Our results indicate that this laboratory-based serologic test can be used to analyze collected sera in epidemiologic studies of *H pylori* infection in children, when its performance has been locally validated.

Several factors may be responsible for the excellent performance of the serologic test in this study. First, results may vary in relation to the gold standard. We used the stool antigen test as the gold standard because it has excellent sensitivity and specificity for children, as compared with gastric biopsy and the rapid urease test.^9^ Second, differences in the performance of ELISA kits are partly due to strain variations. A previous study comparing the performance of the JHM-CAP EIA (an EIA test based on antigens derived from a Japanese strain) and HM-CAP EIA (an EIA test based on antigens derived from a strain in the United States) found that the JHM-CAP EIA had a similarly high specificity and a much higher sensitivity than the HM-CAP EIA.^15^ The higher sensitivity of the JHM-CAP EIA was explained by the presence of a 100-kDa antigen in the Japanese strains, which might be recognized by the host’s immune system at an early stage of infection.^15^ Because the E-Plate EIA also uses strains derived from Japanese patients, we speculate that the sera of Japanese children may exclusively react to the presence of certain antigens in the Japanese strains, such as CagA and VacA. Further studies are needed to explore genetic differences among populations and their effects on immune responses. Third, the validity of the serologic test may be affected by the cutoff points used, given that titers to IgG increase with age in response to *H pylori* infection.^21^ A previous study recommended that, in studies using serologic tests, researchers should reexamine the test results and determine if it is necessary to adopt specialized cutoff points in children.^22^ To address this issue, we evaluated the performance of the serologic test by using various cutoff points and found that cutoff points in the range of 7 to 9 U/ml yielded optimal sensitivity, specificity, and positive likelihood ratio. Although the recommended cutoff point is 10 for adults, cutoff points in the range of 6 to 10 U/ml yield similar values for sensitivity and specificity, according to results provided by the manufacturer. Therefore, our results indicate that no adjustment is needed for Japanese children when the E-plate EIA test is used to detect *H pylori* infection.

Our study has several limitations. First, the samples were collected over a long period, which might have affected the study results. To address this issue, we compared sensitivity and specificity between the earlier (1993–2000) and later periods (2001–2009). Unfortunately, there were too few subjects in the later period, and we could not calculate these indexes. However, the results for the earlier period were similar to those reported for the whole period. Second, because only a subset of children for whom both blood and stool samples were available were included in this study, our results need to be replicated in other, larger samples of consecutive patients. Third, the HpSA test detects infection and current presence of *H pylori*; however, IgG antibodies can be detected approximately 3 weeks after *H pylori* infection.^22^ Therefore, the latent period between *H pylori* infection and antibody production may be a source of misclassification. However, no children under 1 year were found to be positive for IgG antibodies in this study, suggesting that any misclassification derived from the latent period did not bias our results. Finally, we did not collect information, such as number of siblings and birth order, that may be related to transmission of *H pylori* infection among children.

In conclusion, the performance of the E-plate *anti-H pylori* IgG antibody test was comparable to that of the stool antigen test, indicating that it might be useful in epidemiologic studies involving large numbers of participants.
